# Developments in functional imaging of the placenta

**DOI:** 10.1259/bjr.20211010

**Published:** 2022-03-15

**Authors:** Alys Clark, Dimitra Flouri, Nada Mufti, Joanna James, Eleanor Clements, Rosalind Aughwane, Michael Aertsen, Anna David, Andrew Melbourne

**Affiliations:** 1 Auckland Bioengineering Institute, University of Auckland, Auckland, New Zealand; 2 Medical Physics and Biomedical Engineering, University College London, London, UK; 3 School of Biomedical Engineering and Imaging Sciences, King’s College London, London, UK; 4 Elizabeth Garrett Anderson Institute for Women’s Health, University College London, London, UK; 5 Department of Obstetrics and Gynaecology, Faculty of Medical and Health Sciences, University of Auckland, Auckland, New Zealand; 6 Department of Radiology, University Hospitals KU Leuven, Leuven, Belgium

## Abstract

The placenta is both the literal and metaphorical black box of pregnancy. Measurement of the function of the placenta has the potential to enhance our understanding of this enigmatic organ and serve to support obstetric decision making. Advanced imaging techniques are key to support these measurements. This review summarises emerging imaging technology being used to measure the function of the placenta and new developments in the computational analysis of these data. We address three important examples where functional imaging is supporting our understanding of these conditions: fetal growth restriction, placenta accreta, and twin-twin transfusion syndrome.

## Introduction: from biology to the clinic

The placenta is perhaps the most interesting multifunctional animal organ to have ever evolved. The placenta acts as a critical exchange organ for the baby *in utero*, mediating transfer of nutrients and oxygen between mother and fetus whilst keeping their two circulations entirely separate. Placenta-like organs are found across nearly all branches of animals, from primates to sharks, with variable degrees of complexity in anatomy and exchange efficiency.^1,2^ However, the wide variability of placental appearance and structure between species make the human placenta anatomically distinct from many common laboratory and agricultural animal models,^3^ meaning that directly studying the human placenta is critical. *In vivo* imaging of the placenta in human pregnancy is thus a valuable tool for understanding normal and abnormal placental function.

**Figure 1.**
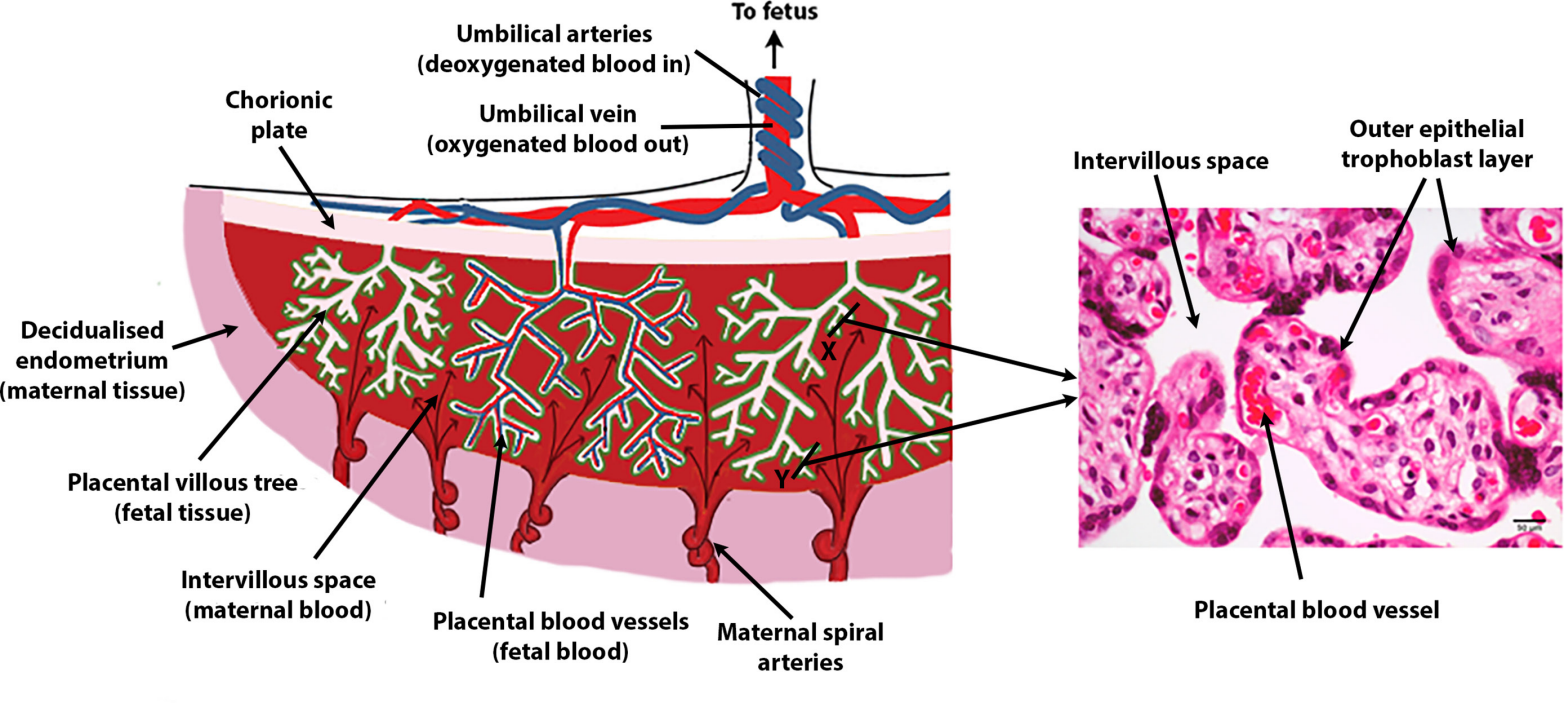
Illustrations of human placental features and cellular relationships.

The human placenta is disc-shaped and weighs around 500g at term. Indeed, ‘placenta’ is the latin term for ‘flat cake’, and the size and shape are not dissimilar. Structurally, what initially appears as a relatively solid disc is in fact comprised of extensively and densely branched villous trees, which branch from the chorionic plate (the aspect of the placenta closest to the fetus).^4^ These villous trees are grouped into discrete amorphous functional units termed lobules (or occasionally cotyledons) of which 20–25 are ordinarily visible in the human placenta. For most of pregnancy, maternal blood flows from the uterine circulation and circulates around the outside of these villous trees. Exchange then occurs across the outer epithelial layer of the placenta, which has a surface area of 12 m^2^ by the end of pregnancy.^4,5^ Within the placental lobules, a complex-branched fetal vascular network carries deoxygenated blood from the fetus to the exchange surface with the mother, and returns oxygenated blood back to the fetus via the umbilical vein (Figure 1).

Over the course of pregnancy, the placenta extensively remodels the maternal uterine vasculature, to facilitate a 15-fold increase in blood flow to the placenta.^6^ This increase in nutrient and oxygen supply is crucial for adequate fetal growth. To achieve this, specialised placental cells (called trophoblasts) migrate out from the placenta and invade the endometrium, the inner layer of the maternal uterine cavity and beyond, up to the level of the uterine muscle or myometrium. Once in this tissue the trophoblast cells invade into glands, veins, and lymph vessels of the uterine wall to provide nutritional support of the embryo with substances in maternal plasma and uterine gland secretion products. The trophoblast cells replace the muscle in the walls of the terminal spiral arteries and remodel them eventually into wide non-vasoactive tubes that open out to supply blood to the placenta.^7^ Hormones and other paracrine factors secreted by the placenta act to remodel the larger upstream vessels, which double in size by mid-gestation.^8^ Together, these changes enable a higher volume of blood to be delivered to the placenta, but at an appropriate velocity of flow around the placental villous tree to optimise exchange efficiency.

The successful growth and development of the placenta, and its adaptation of the maternal uterine circulation are critical for pregnancy success. However, inadequacies in multiple points across this system can occur, resulting in pregnancy disorders such as pre-eclampsia (when inflammatory signals from the placenta cause dangerously high maternal blood pressure), or fetal growth restriction (FGR, when the fetal growth rate decelerates or stagnates *in utero*) due to placental insufficiency.^9–11^ Conversely, abnormal placental attachment can occur at the site of scars from previous caesarean section or other uterine incisions or trauma. This condition termed placenta accreta spectrum can lead to life-threatening maternal haemorrhage at the time of delivery, as well as the need for hysterectomy to completely remove the invasive placenta.^12^ Identical twins and higher multiple pregnancies also create unique scenarios *in utero* due to vascular connections within the shared placenta, potentially resulting in discrepancies in blood distribution between the fetuses called twin-to-twin transfusion syndrome (TTTS) that can be life-threatening for both if not treated *in utero*.^13^ As a result, the placenta is a clinically important target for developments in imaging technology, so that scientists and clinicians can examine the structure and function of the organ *in utero* and improve maternal and neonatal outcomes.

This review highlights some common imaging technologies used in pregnancy, with a focus on new developments to quantify placental function from imaging.

## What is being used at the moment to measure placenta function?

### Ultrasound

**Figure 2.**
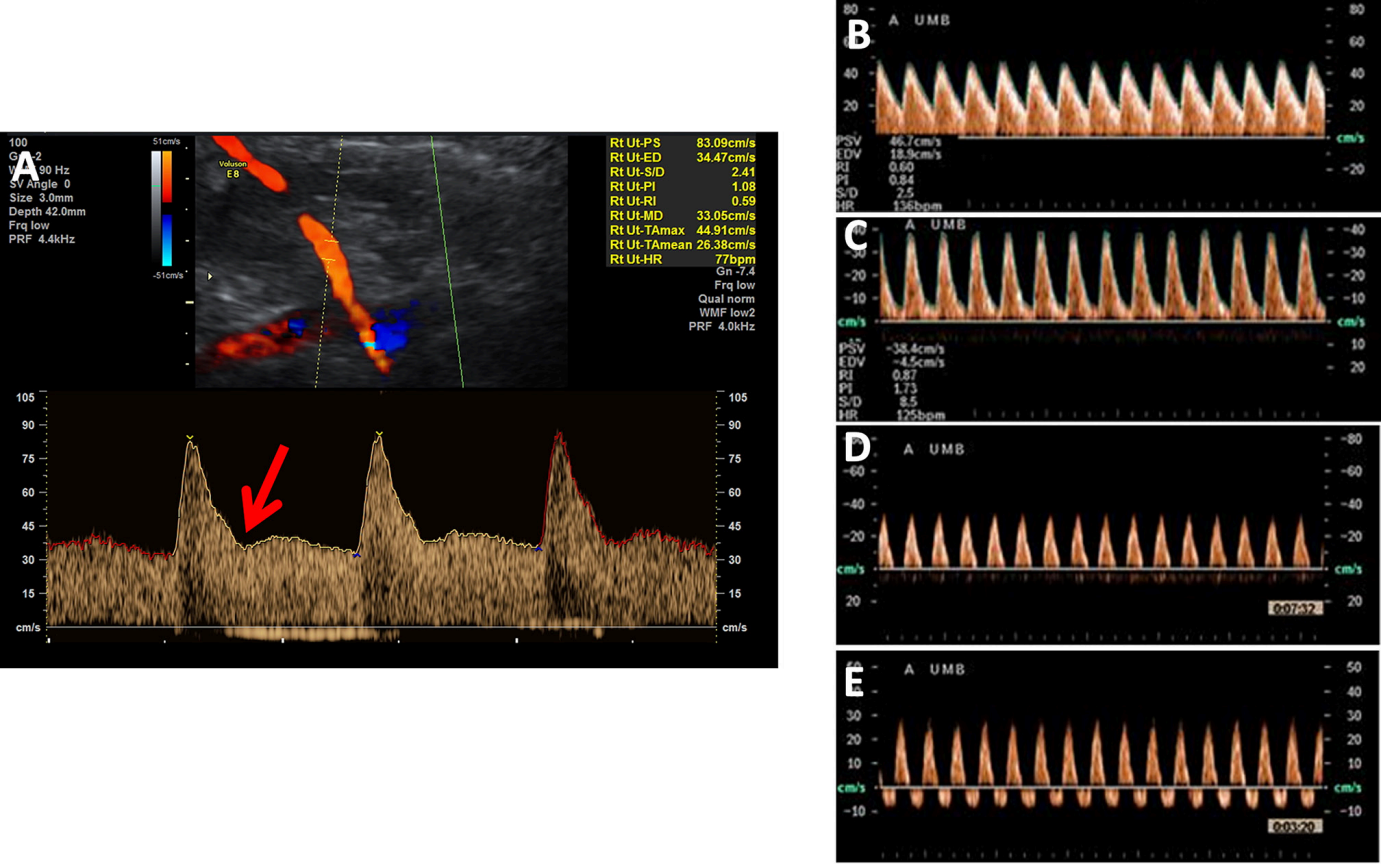
a: Prediastolic notching of the uterine artery waveform (red arrow) and raised pulsatility index (PI) at 22 weeks indicates placental insufficiency and has a high positive predictive value for fetal growth restriction b: Normal umbilical artery Doppler Increasing resistance in the umbilical artery leads to the development of raised PI (c), absent (d) and then reversed end diastolic flow (e).

Ultrasound is the most routinely used clinical imaging tool for investigation of the placenta *in vivo*. Obstetric ultrasound typically assesses fetal growth through serial fetal measurements and placental shape, cord insertion and uterine position using gray scale. Pregnancies commonly have placental blood flow inputs and outputs monitored via assessment of uterine, umbilical, fetal middle cerebral arterial and ductus venosus Doppler ultrasound flow waveforms (Figure 2). Doppler ultrasound provides a functional interpretation of the blood flow in these vessels, inferring information on placental perfusion and fetal wellbeing by the shape, direction and magnitude of their flow velocity. In chronic hypoxia, the fetal circulation is redistributed towards the brain away from other circulatory beds, especially to the fetal kidneys and lower limbs leading to increased middle cerebral artery perfusion.

Maternal placental perfusion can be estimated *in vivo* with measurement of uterine artery volume blood flow, pulsatility and resistance indices. The uterine arteries vasodilate from early in normal pregnancy, providing increased volume blood flow to the uterus and developing fetus. Doppler analysis of the uterine arteries from the first trimester can indicate a poorly developed utero-placental circulation, leading to placental insufficiency and fetal growth restriction (FGR). Typical sonographic findings include an increased pulsatility index and pre-diastolic notching in the uterine artery Doppler waveform indicating high vascular resistance. There is a well-established association between increased uterine artery pulsatility index in the late second trimester, and the development of early onset pre-eclampsia and FGR,.^14–16^ However, the sensitivity and specificity are poor.^17^

The fetoplacental circulation can be examined *in vivo* with umbilical artery Doppler measurements and is used clinically to give an indication of fetal wellbeing.^18^ An increased umbilical artery pulsatility index and reduced or reversed end-diastolic flow indicates increasing resistance in the fetoplacental vascular bed, as seen in placental insufficiency.^19,20^ This relationship has been validated in animal models, where progressive embolisation of fetal vessels in sheep resulted in progression from normal to absent to reversed end diastolic flow in the umbilical artery.^21^

Redistribution of the fetal cardiac output in response to developing fetal hypoxia can also be detected antenatally, via a reduction in the middle cerebral artery (MCA) pulsatility index indicating cerebrovascular dilatation^22,23^

While not yet used clinically at this stage, more detailed Doppler analyses using power or colour Doppler are beginning to be used to produce detailed maps of the circulation at the utero-placental interface^24,25^ and in the feto-placental circulation.^26^ These emerging ultrasound techniques interpret the ultrasound scatter due to the presence of red blood cells to provide maps representing blood flow velocity (colour Doppler) or speed (power Doppler). They may provide more detailed functional interpretation than their predecessors, but care must be taken to ensure that machine settings are comparable when interpreting these data,^27^ and so ensuring consistency and reproducibility in methodologies, and significant validation is required before routine clinical use. As a result, clinical ultrasound relies heavily on morphometric rather than functional imaging whilst analysis of Doppler waveforms can be susceptibility to intrasubject variability and the use of compound measurements involving ratios.

### MRI

MRI is safe in pregnancy^28,29^ and the whole placenta may be imaged at any gestational age. Although many new techniques have been attempted, the use of MRI in the assessment of placental function in the clinic is not widespread. The reasons for this are complex, but the promise of MRI is to provide quantitative measurement of placental function. At this stage, many MR techniques have been proposed, but very few, if any, have been validated with a causal mechanism that supports the correlations observed.^30^ Like emerging ultrasound technologies, this validation is critical for clinical adoption, and this absence of physiologically grounded knowledge is restricting placental MRI from further integration in maternal-fetal medicine.

Dynamic contrast-enhanced magnetic resonance imaging (DCE-MRI) is an imaging technique that enables spatial and quantitative characterisation of the maternal perfusion in normal or pathological conditions such as placental insufficiency with an injection of contrast agent.^31–34^ DCE-MRI is performed by using fast imaging sequences (*e.g.,* gradient echo) that are repeatedly applied over the organ of interest during bolus administration of a contrast agent. The temporal uptake of contrast agent measured from the image time-series provides quantitative information about tissue blood perfusion. One of the first studies in which gadolinium contrast was used to assess human placental function in second and third trimester was performed by Marcos et al..^35^ The diagnostic potential of DCE-MRI has also been assessed in placenta accreta spectrum (PAS) disorders (placenta previa and abnormal invasive placenta).^32^ In this study DCE-MRI allowed the extraction of tissue enhancement parameters of the uterus and materno-placental circulation that differed significantly between pregnancies with PAS and normal pregnancies. DCE-MRI has also been used in animal models.^36–39^ Previous studies in mice have shown differences in placental perfusion between normal and disease conditions at a given gestation.^40–43^ More recent studies have showed potential in detecting abnormal flow patterns in placentas affected by fetal growth restriction whilst ruling out PAS.^44^ Despite relatively slow trans-placental transfer, DCE-MRI clinically is limited by the use of exogenous contrast agents based on gadolinium which have an unknown safety profile in pregnancy. Contrast agents may cross to the fetus and are recirculated in the amniotic fluid and questions remain about long term accumulation of gadolinium.^28^ As a result, it is only recommended for clinical use if it significantly enhances diagnostic performance and is expected to improve fetal or maternal outcome such as in the case of PAS.^45^ This motivates the development of non-contrast agent-based techniques such as diffusion imaging to measure placental function.

An alternative to DCE-MRI for measuring the properties of the maternal circulation is Arterial Spin Labelled MRI. This technique makes use of magnetic pre-labelling of blood as it passes into the field of view. As a result, this technique is free of exogenous contrast agents. Blood flow is measured by subtracting labelled and un-labelled images and as a result this technique is susceptible to motion artefacts between images which can corrupt the result. Additionally, the relatively low SNR requires high field strength and multiple averages to be acquired. Despite this, the technique has potential in the placenta to reveal the properties especially of the materno-placental circulation^46–49^ and when combined with sophisticated image analysis techniques, the extracted parameters have the potential to be quite robust.^50,51^

**Figure 3.**
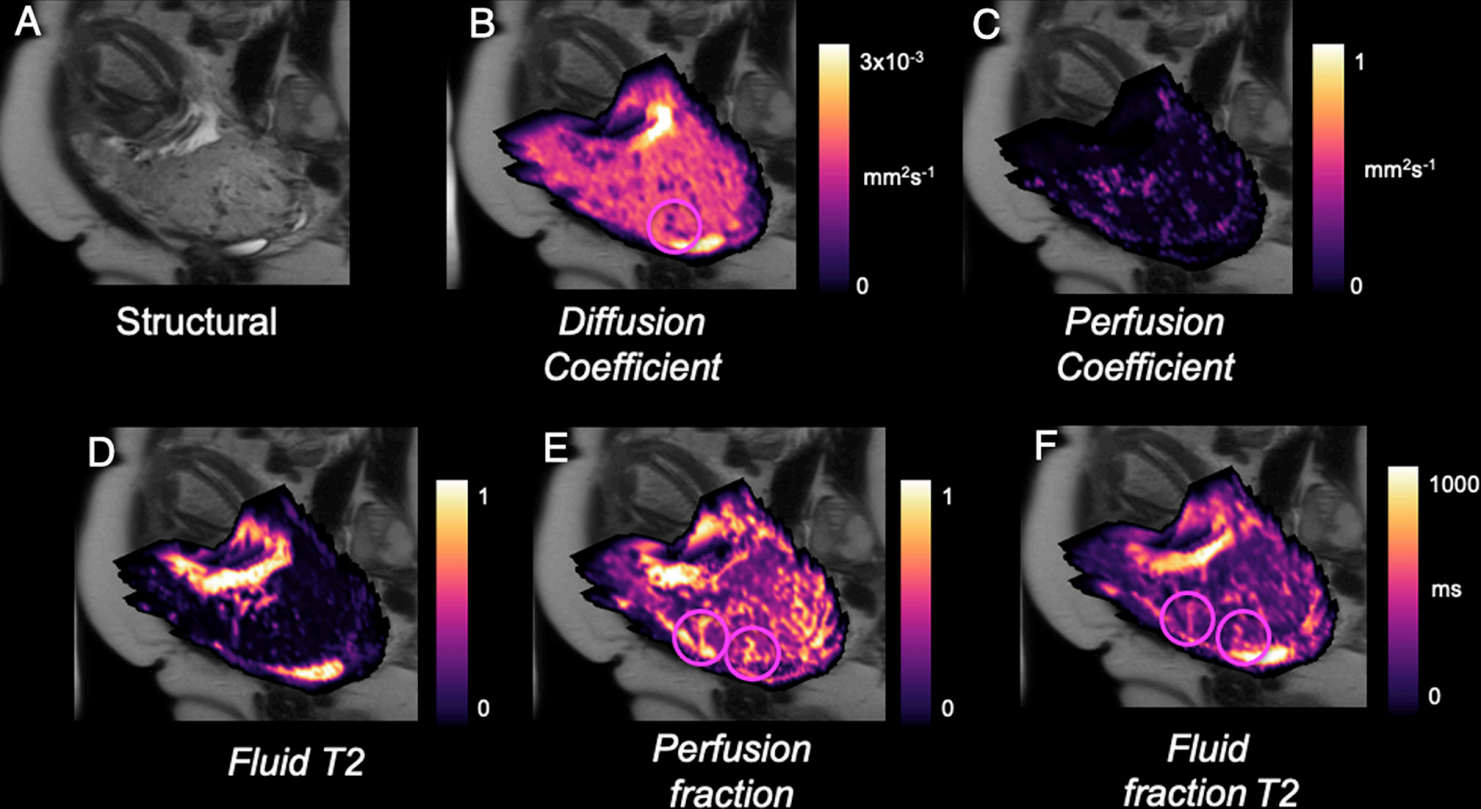
Example parametric maps of placental function from MRI in an example of PAS. a) T2-weighted structural image, b) apparent diffusivity of placenta c) Pseudo-diffusivity of placenta[53,68] d) Placental T2[69], e) Placental perfusion and free-fluid signal [52]. Circles highlight vascular features close to the placental-myometrial border.

Diffusion-weighted (DW) MRI uses the random motion of water molecules within tissue as contrast, providing information on diffusion within placental tissue and the exchange properties of the maternal and fetal circulations.^52^ In DW-MRI, perfusion can be approximated using intravoxel incoherent motion (IVIM) model.^53,54^ In IVIM-DW imaging, perfusion is evaluated by exploiting the fact that blood in each voxel has pseudorandom translational motion within the capillaries, providing access to perfusion when using small magnetic field gradients during the MR pulse sequences. Placental diffusion and perfusion changes measured with IVIM-MRI technique have been gaining recognition^55–57^ (Figure 3). A recent study demonstrated the effect of maternal sleep position on utero-placental and feto-placental blood flow oxygenation in healthy late gestation pregnancy using DW-MRI.^58^ Diffusion-tensor (DT) MRI, an extension of DW-MRI, computes diffusion anisotropy. Fractional anisotropy could be detected by DT-MRI to differentiate functional placental tissue.^59^

The sensitivity of T2* to oxygenation and the rapidity of acquiring a T2*-weighted gradient echo image mean that oxygen flux can be monitored dynamically. This technology was originally developed in the context of investigating functional changes in perfusion and oxygen usage in the human brain in fMRI.^60–65^ However, it also has important applications in pregnancy and has been shown to be sensitive to changing placental oxygen levels during uterine contractions and external changes to the maternal oxygen level.^66,67^

Although MRI technology shows promise detecting placental abnormalities, it has not been adopted yet in clinical practice likely due to several challenges in accessibility. Both examination costs and comfort play a role in the relatively limited availability of studies on MRI of human placental function and, despite the rich range of contrasts available, both MR structural and temporal resolution is comparably low compared to ultrasound. If these challenges can be overcome, MRI could be a valuable method to detect abnormal placental function in clinical practice.

## What clinical questions could measurement of placental function answer?

The shape and structure of the feto-placental vasculature has been linked to a number of pregnancy pathologies. Changes are seen throughout the vasculature ranging from the largest chorionic blood vessels that are considered the framework upon which the entire placental vascular tree is built, down to the smallest capillary blood vessels.^68–71^ This disrupted vasculature is evident in pre-eclampsia, FGR,^72–75^ diabetic pregnancies,^76^ placenta accreta^77–79^, preterm premature rupture of membranes and identical twin pregnancies.^80–82^ Measurements of the vascular topology and function will help us to model how the placenta is functioning in these critical pathologies.

### Application to Fetal Growth Restriction (FGR)

Abnormal placental function leads to FGR and maternal hypertensive disease. Preventing FGR or its early identification reduces the risk of stillbirth and has the potential to improve lifelong health. Ultimately poor placental function results in sustained nutrient restriction and fetal hypoxia, which can lead to long-term neurocognitive and cardiovascular impairment in the child and adult.^83,84^ But even low birthweight (<2.5 kg) is associated with dramatically increased risks of cardiovascular and metabolic disease in later life.^85,86^ Antenatal identification of abnormal placental function prior to development of fetal and maternal sequelae is therefore a health priority, but developing technologies to achieve this has proved challenging. International advances in placental imaging now provide the realistic prospect of detecting abnormal function early enough in gestation to allow treatments to be administered to those at risk. Novel treatments are currently being developed with the potential to treat placental insufficiency, so developing better ways to precisely measure changes in placental function is a priority.

Clinical care is currently guided by assessment of maternal risk factors, measurement of uterine artery blood flow using Doppler, monitoring fetal growth and fetal heart rate variability using antenatal cardiotocography (CTG). Maternal circulating proteins such as the growth factors PAPP-A and PlGF^87,88^ are insufficiently sensitive and specific to predict placental insufficiency and FGR alone, and small fetal size is not equivalent to fetal growth restriction; fetuses whose estimated fetal weight is within the normal range, but that have placental insufficiency with reduced growth velocity can easily be missed. These gaps in our knowledge mean that care in these pregnancies may not be optimal. The ability of MRI to detect differences in the placentas of pregnancies with early onset FGR (<32 weeks of gestation) associated with placental insufficiency is well established.^75,89,90^ As yet unknown is the ability of imaging to measure placental insufficiency more broadly in circumstances where anatomical differences in feto-placental circulation are more subtle, but the effects of resulting chronic hypoxia may still be clinically important although less easily detected.

There are no known risks to the fetus from MRI, including within the first trimester,^28,29^ and the whole placenta may be imaged at any gestational age, something that is not possible to achieve using ultrasound in the second half of pregnancy. Whilst also falling with gestational age,^91,92^ T2 and T2* relaxation time, which relate to the structure and oxygen level of the tissue, decrease substantially in placental insufficiency compared to normal placentas.^66,93^ The impedance of diffusion of water molecules, and thus oxygen, is increased in placental insufficiency compared to normal placentas^94,95^ and the vascular perfusion fraction and oxygenation, measured using diffusion imaging with placenta-specific modelling, is reduced in placental insufficiency compared to normal placentas.^75,96–98^

Late stillbirth is fortunately uncommon (3.9 per 1,000 UK births) but is more frequently found in the situation of undetected late onset FGR where the fetus may be only slightly growth restricted. It is now well recognized that maternal supine sleep position in late pregnancy is independently associated with an increased risk of stillbirth,^99^ and all females are recommended to go to sleep on their side-in the third trimester of pregnancy. The pathological mechanism leading to stillbirth is thought to be the gravid uterus compressing the inferior vena cava (IVC) when a female lies in the supine position during late pregnancy.^100^ Using MRI, it has recently been shown that compared to the left lateral position, maternal supine position in healthy late pregnancy is associated with reduced utero-placental blood flow and oxygen transfer across the placenta. There was an average 6.2% reduction in oxygen delivery to the fetus and an average 11% reduction in fetal umbilical venous blood flow.^58^ Thus even in healthy late gestation pregnancy, maternal position significantly affects oxygen transfer across the placenta and may partly explain late stillbirth in vulnerable fetuses.

After these findings, the next steps are 1) to validate physiological markers from MRI by making use of pre-clinical studies and 2) to broaden clinical studies to broader phenotypes of placental insufficiency such as late onset FGR (>32 weeks of gestation), and females with reduced fetal movements. This will allow the assessment of less severe, but more complex phenotypes where current clinical tools to assess fetal wellbeing such as doppler ultrasound and CTG are limited and further allow a link to be established between MR parameters and early changes in placental function in human pregnancy and generate evidence-based hypotheses about the defined pathological changes in the placenta observed in humans. Future work in this area will improve our understanding of placental pathophysiology and advance the usefulness of imaging in caring for females and their babies in pregnancy.

### Application to placenta accreta

Placenta Accreta Spectrum (PAS) Disorders involve abnormal placental adherence and vascular disruption to the myometrium, leading to life-threatening maternal haemorrhage.^101,102^ The condition has a prevalence of 0.04–0.42% pregnancies.^103^ However, the incidence of PAS rises with successive caesarean section deliveries, with a rate of 4.1% in females with one prior caesarean delivery and 13.3% in females with two or more previous caesareans.^104^ As the rate of caesarean section has reached>50% in some countries, the optimal diagnosis and management of PAS pregnancy is becoming critical in obstetrics.^105^

**Figure 4.**
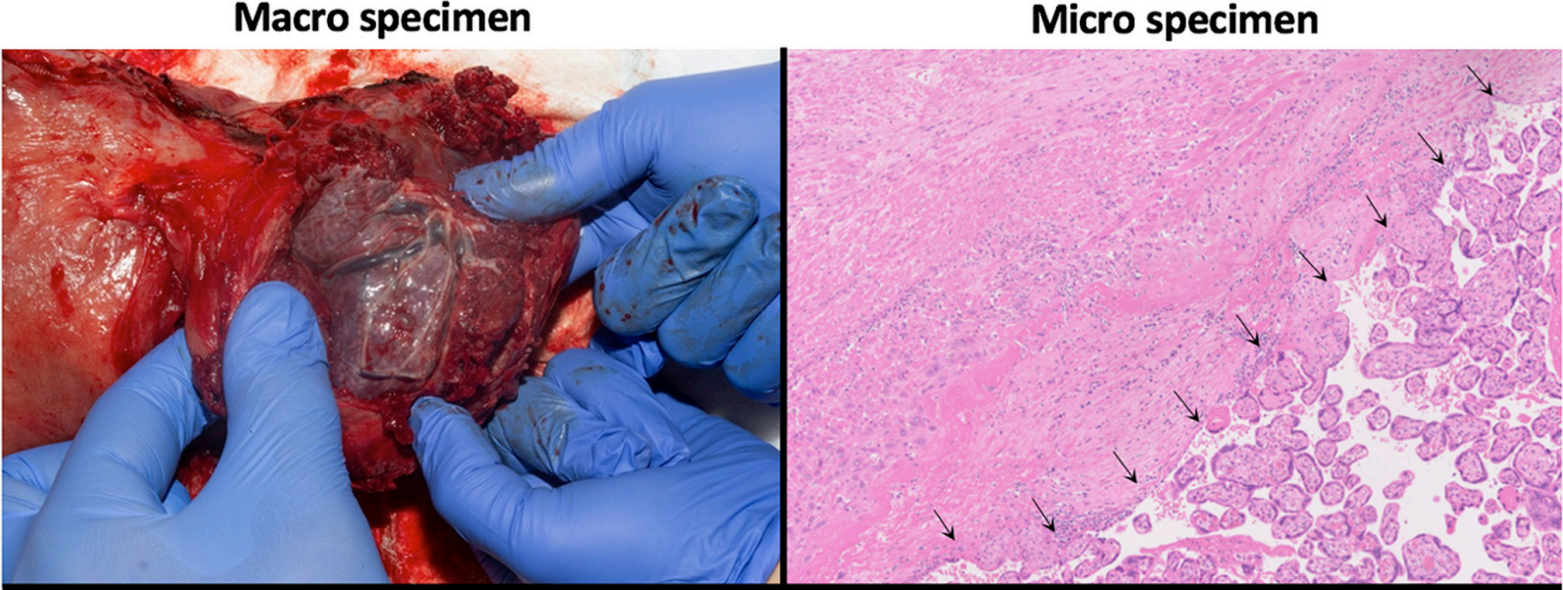
Macro (left) and Micro (right) specimens for a PAS disorder patient. Arrows show: Chorionic villi directly related to the myometrium with no intervening decidua. Appearances are in keeping with placenta accreta.

**Figure 5.**
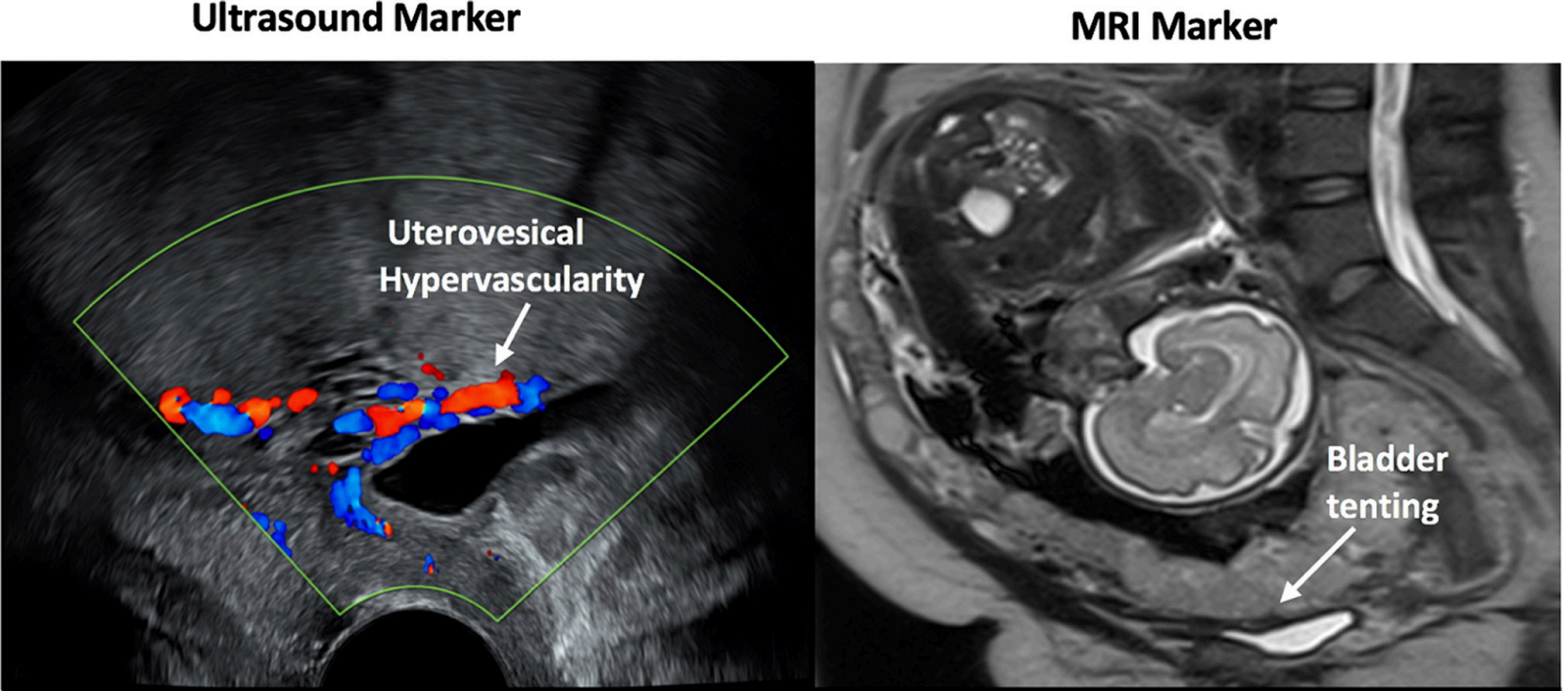
Examples of morphological ultrasound (left) and MRI (right) markers in a patient with PAS disorder confirmed on histopathology of the caesarean hysterectomy specimen. The imaging signs are labelled within the figure.

**Figure 6.**
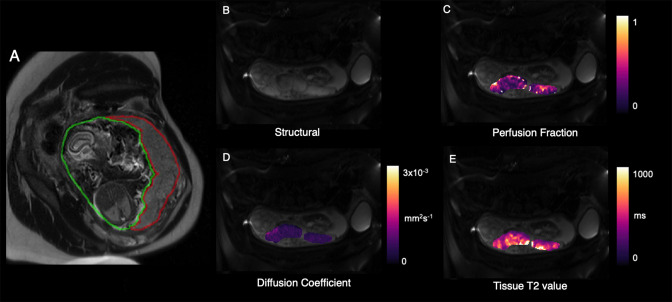
Example parametric maps of placental function from MRI in an example of TTTS. a) T2 weighted structural image and whole placenta segmentation (red), b) Structural image with no diffusion weighting, c) Placental perfusion^52^,d) apparent diffusivity of placenta and e) Tissue T2 value^93^

The placenta is separated from the myometrium by the decidua.^106^ Injury to the endometrium, through uterine surgery such as caesarean section, can result in a decidual deficit in subsequent pregnancies causing abnormal placentation whereby chorionic villi directly abut the myometrium and extra villous trophoblast invasion is increased.^107,108^ A histopathological specimen of a placenta accreta case is shown in Figure 4. Failure to recognise and manage PAS disorders at delivery can lead to massive post-partum haemorrhage with a mortality rate of 2.6–7% as the placenta fails to detach from the uterine wall and surrounding tissues.^109^ It is important to correctly diagnose this disorder, with accurate assessment of the extent of abnormal attachment for better surgical planning. This is currently being performed by subjective interpretation of typical sonographic markers using 2D grey-scale and Doppler imaging, with MRI only used as an adjunct.^101,110^ A standardised protocol for ultrasound reporting of findings (*e.g.,* abnormal placental lacunae, or myometrial thinning) has been published by the European Working Group on Abnormally Invasive Placenta (EW-AIP).^111,112^ These findings have a varying degree of sensitivity and specificity.^110^ Similarly, some structural MRI signs include dark T2 intraplacental bands, placental heterogenous signal intensity, uterine bulging, focal interruption of the myometrium and tenting of the bladder.^108,110^ An example of these radiological signs is illustrated in Figure 5. These morphologic markers are highly subjective, even when functional MRI methods such as DCE, DWI, and IVIM are employed^32^ and have varying sensitivity and specificity. Recently standard imaging protocols, reporting terminology and structured reports have been proposed. This should lead to improved reporting and comparison between different studies and techniques.Figure 6 ^113–115^

The limitation of these methods includes low spatial resolution and difficulty assessing vascular invasion.^32,116,117^ Combined T2 relaxometry (T2R) and intravoxel incoherent motion (IVIM) signal models can be used to separate signals from fetal and maternal blood pools over a region of interest of previous scar tissue and suspected abnormal placentation.^52,90,118^ By studying how parameters from this multicompartmental model vary in PAS disorders it may be possible to identify differences in vascularity and perfusion for an objective quantification of abnormal placentation and to measure the effects of abnormal trophoblast invasion. Similarly, advances in image reconstruction technology^119,120^ applied to areas of abnormal attachment, may better distinguish between degrees of abnormality and thus support clinical decisions for delivery.

### Application to Twin-twin transfusion syndrome

Around one-third of twin pregnancies in the UK share a placenta. The mortality rate associated with these twins is 11%, with 44% of this mortality caused by a condition known as Twin-to-Twin transfusion syndrome (TTTS), which results from an interfetal transfusion imbalance because of placental chorionic vascular anastomoses.^121^ TTTS manifests as fluid overload in the recipient twin and fluid depletion in the donor.^122^ Untreated TTTS is associated with the death of one or more fetuses in more than 80% of pregnancies^123,124^ and is accompanied by high rates of morbidity in surviving fetuses.^125^ Current treatment via fetoscopic laser ablation (FLA) of selected chorionic vessels is supported by level one randomised control trial data.^126^ A modification to the technique known as the Solomon technique, involves coagulation of the vascular equator of the chorionic surface after selective FLA. This has now been widely accepted as being more effective, but damages a larger proportion of the shared placenta.^127^ A further condition called Twin Anaemia Polycythaemia Sequence (TAPS) can also develop due to a discordance in haemoglobin levels. This may also be treatable using FLA.^128^

Ultrasound plays a vital role in the management of multiple pregnancies with a shared placenta starting with a correct determination of the chorionicity in the first trimester to ensure more intensive surveillance of high risk pregnancies.^129^ In the following weeks ultrasound remains crucial in the detection of complicated fetal outcomes from 16 weeks onwards in view of potential fetal therapy.^130–132^ Although ultrasound helps in the detection of TTTS,^133^ it remains impossible to measure placental blood sharing accurately in these pregnancies and therefore the progression of the severity of TTTS is currently unpredictable. Careful follow-up is therefore required because of the higher chance of sudden and significant intertwin transfusion imbalances after 26 weeks in a twin pregnancy with growth discordance.^134^ Furthermore, although TTTS occurs mainly before 26 weeks, it can occur at any time during a continuing twin pregnancy.^135^

Improving outcomes relies on more detailed pre-surgical planning, predicting the effect of different formations of connecting vessels and modelling changes in deep placental function as these changes are made. Advanced MRI methods have the potential to extract combined information relevant to blood redistribution in TTTS.^46,52,69,136^ In the future, MRI models could provide a mapping of vessel anastomoses and vascular density to establish correspondence between *in vivo* appearance and function and to make computational predictions based on pre-surgical MRI. Such a computational model would allow to predict the intraoperative effects of laser vessel ablation on the flow dynamics of twin placentae during intervention.

Several core imaging technologies have been developed recently that have potential for enhancing surgery in TTTS. For instance, advanced feto-placental super-resolution reconstruction and segmentation^120,137–139^ may be adapted to allow visualization and measurement of the placental chorionic vascular tree.^70,140,141^ This is essential information prior to laser ablation of the vascular anastmomoses. Furthermore, advances allow measurement of placental structure and function using MRI^52,142,143^ ; and recent techniques for blood flow simulation allow placental chorionic vessel blood flow modelling.^141,144^ Combined these techniques could provide prediction of the haemodynamic response during surgery.

Post-delivery, the morphology of the placenta can be assessed and compared with outcome using several protocols.^70,140,145^ High-resolution microCT data enable a high-resolution reconstruction of the placental vascular structure for computational flow modelling without affecting subsequent placental histology.^145^ This modelling may allow the prediction of how conditions such as TTTS and TAPS can develop from certain configurations of placental anastomoses.

## Looking forward: Mixed models and computational modelling

### Combining data from MRI

The power of recent MRI models arises from the ability to combine information from different sources of contrast. Several attempts have been made to use combinations of MRI sequences to disentangle information about placental perfusion and oxygenation.^146–148^

Imaging from MRI typically is used to represent a single parameter of interest such as T2 or diffusivity.^91,93,149^ By acquiring acquisitions across multiple contrasts, it becomes possible to isolate the contribution to the T2 or diffusivity from separate placental sources,^147,148,150^ for instance to measure properties of the maternal and fetal circulations separately. This approach has been used in FGR^92^ and to analyses the effect of maternal position on placental function.^151^ Attempts have also been made to carry out this separation using ultrasound.^152^ Current interest in uterine contractions and the effect on placental function suggests that the combination of information from dynamic BOLD MRI could be combined with markers of volume or shape change in the placenta.^63,153^ This approach could enhance our knowledge of the impact of uterine contractions before and during labour, allow the derivation of information on placental capacity and uterine power, and help to provide useful information prior to delivery.

Despite promise, these approaches often require offline processing and significant computational power to produce novel parameter maps presenting quantitative information back to the clinician but have the potential to bring new markers to the clinic.

### Computational modelling

While MRI (and other imaging modalities) can assess structural and functional changes between individuals and populations with different pregnancy complications, the multifactorial contributors to fetal health during pregnancy mean that additional insights into the connection between placental structure and function could be invaluable to interpreting imaging going forward.

One strategy to achieve this is via mathematical and computational modelling of placental structure and function. Interpretation of MRI has long been guided by mathematical models, for example, compartmental models of tissue/blood in diffusion weighted imaging. However, personalised modelling approaches are emerging that allow for anatomical data in an individual to be used to predict their placental function. These models could potentially be used to improve analysis of acquired MRI images, and to test hypotheses around what drives function in an individual pregnancy or group of pregnancies, better enabling identification of target “features” in imaging. There are a number of studies that have assessed the morphology of the post-delivery placenta in 3D, with micro-CT imaging emerging as a useful tool to do this.^70,71,154^

Whole organ models of the placenta have emerged that aim to describe how blood is distributed throughout the placenta, given the anatomical structure of the blood vessels (size and 3D distribution) that reside within it.^144,154,155^ These models treat individual blood vessels as elements that are resistive to flow, and this allows an electric circuit analogy to be applied to predict the distribution of flow and blood pressure within the system. As has recently been demonstrated in rodent models, this analogy can be taken further to predict functional Doppler resulting from the anatomical structure of blood vessels within an organ.^156^ This type of modelling provides an opportunity to provide new knowledge on how clinical ultrasound, MRI and functional anatomy relate. Byrne et al^154^ demonstrated how variation in anatomy, even at the larger chorionic artery level, leads to significant heterogeneity in blood flow distribution in the feto-placental unit. This heterogeneity was proposed to relate to the capacity of the placenta, i.e. a placenta may function normally, but the heterogeneity in its function may place the placenta at higher risk of a loss of function if part of the placenta experiences a pathological impact. Heterogeneity in structure and function has been observed across MRI studies, particularly in fetal growth restriction, and computational modelling provides an avenue to interpret this heterogeneity in function in terms of risk to fetal blood or oxygen^157–163^

**Figure 7.**
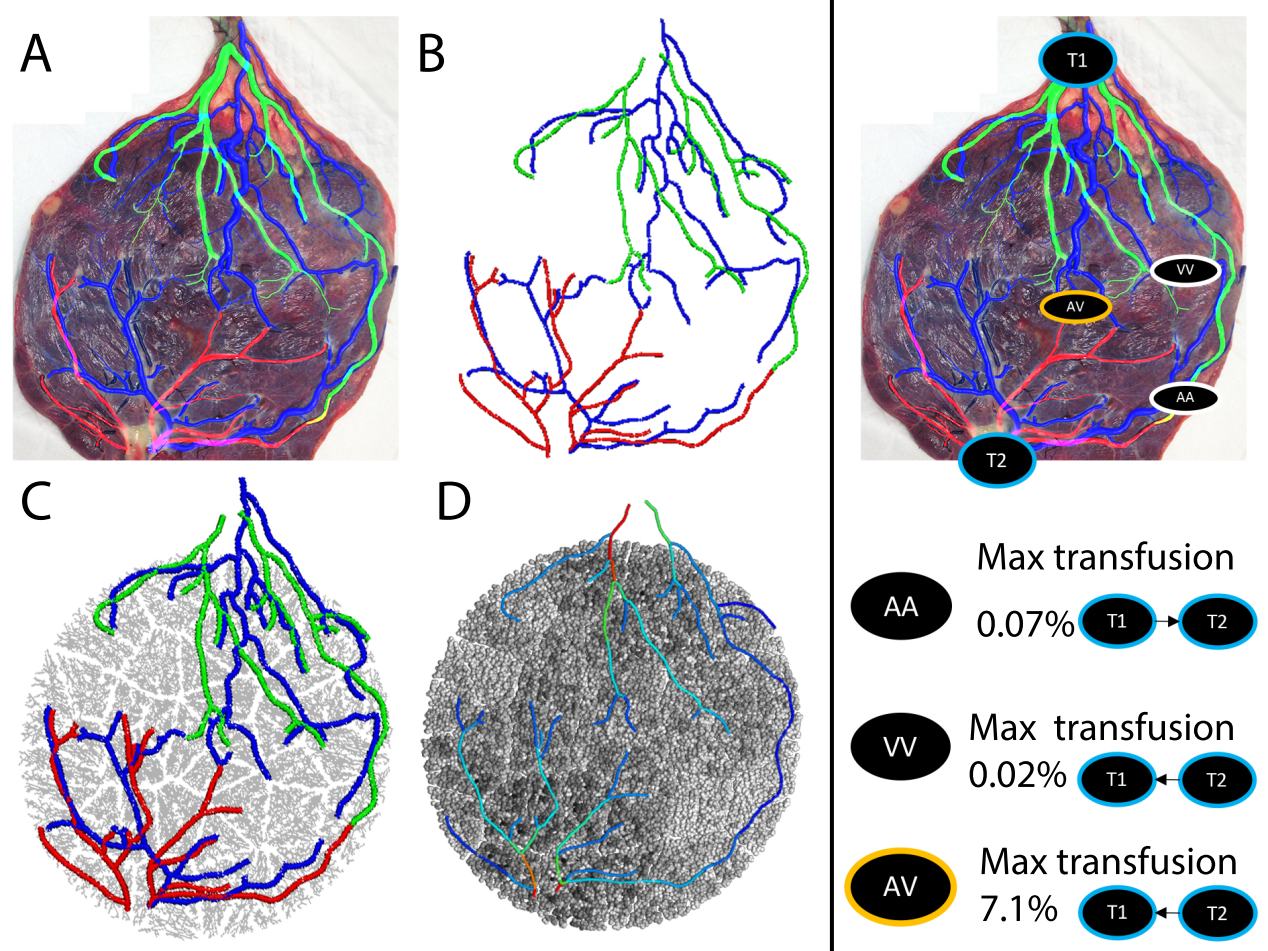
A computational model of a twin placenta derived from a map of arteries and veins on the placental chorionic surface. Left panel: (**a**) Arteries and veins are segmented and (**b**) their centerlines are converted to a series of connected nodes and elements (in a graph-like structure)[71]. (**c**) this graph like structure is mapped to a 3D model of the placenta derived from an ellipsoidal fit to the placental boundary[143] (Clark et al. 2015). (**d**) Blood flow can then be simulated in the major chorionic vessels (colourmap red = 150 ml/min, blue = 1 ml/min) as well as in the gas exchange tissue of the placenta (colourmap black = 0.05 ml/min, white = 0 ml/min). Right panel: Visible arterio-arterial (AA), veno-venous (VV) and arterio-venous (AV) anastomoses can be visualised and be included/removed from the model systematically to assess their individual impact on twin-twin transfusion. In this example, the AA and VV anastomoses have a small contribution, but the AV anastomosis is predicted to transfuse up to 7% of blood flow from twin 2 (**T2**) to twin 1 (**T1**). Note that this placenta is from a pregnancy with normal outcome and does not have a clearly identified AV anastomosis, so the addition of this connection is artificial.

To illustrate how computational modelling could be used to guide surgical planning in TTTS, the model of Byrne et al^69^ is applied to assess the chorionic vasculature of a mono-chorionic twin pregnancy in Figure 7. In this case chorionic arteries and veins were derived from photographs of the placenta obtained after delivery, however, these maps could potentially be derived in treatment planning from ultrasound or MRI [.^122,136,137^ In this type of model, segmented vascular maps are converted to a graph-like network of nodes and elements, within which blood flow is simulated using Poiseuille’s Law along with conservation of mass at bifurcations.^144^ The distribution of blood flow between the two placentas can then be simulated in the absence of any anastomoses, and then in a systematic manner by adding and removing anastomoses to the model. There are three types of anastomoses to consider: true arterio-arterial (AA) anastomoses and veno-venous (VV) anastomoses allow immediate and bi-directional flow and pulse propagation between the circulatory systems of each twin; arterio-venous (AV) anastomoses are implicitly uni-directional, the arterial supply from one twin drains into the venous output of the second, implying that the placental tissue in these instances is shared between twins.^121,164^ In this example, the imaged placenta contained arteries and veins from each twin that came in close proximity, but with no anastomosis visible on the choronic surface, hence the AV anastomosis applied was artificial. Model predictions are consistent with the hypotheses that the emergence of TTTS or TAPS is linked to a relative imbalance in the number and size of these anastomoses; net transfusion from AV anastomoses is not compensated by true AA or VV anastomoses^165–167^ The strength of the use of computational models in this area is in their ability to assess this net transfusion from anatomical data that may be available in surgical planning on a case-by-case basis.

## Conclusion

This review has presented a summary of current functional imaging techniques for the placenta and some of the computational imaging technology being used to extract individualised placental models. These are likely to play a growing role in imaging developments, although we are still some way from the validation required to bring them to the clinical setting. The potential for improving our understanding of the most interesting of organs and the ability to advance clinical management make this an exciting time for placental research.
